# Discovery and Proof-of-Concept Study of Nuclease Activity as a Novel Biomarker for Breast Cancer Tumors

**DOI:** 10.3390/cancers13020276

**Published:** 2021-01-13

**Authors:** Luiza I. Hernandez, Marcos J. Araúzo-Bravo, Daniela Gerovska, Ricardo Rezola Solaun, Isabel Machado, Alien Balian, Juliana Botero, Tania Jiménez, Olaia Zuriarrain Bergara, Lide Larburu Gurruchaga, Ander Urruticoechea, Frank J. Hernandez

**Affiliations:** 1 SOMAprobes S.L, Science and Technology Park of Gipuzkoa, 20009 San Sebastian, Spain; luiza.hernandez@somaprobes.com (L.I.H.); isabel.machado@somaprobes.com (I.M.); jbotero001@ehu.eus (J.B.); tania.jimenez@somaprobes.com (T.J.); 2 Department of Cell and Developmental Biology, Max Planck Institute for Molecular Biomedicine, 48149 Münster, Germany; marcos.arauzo@biodonostia.org; 3 Computational Biology and Systems Biomedicine, Biodonostia Health Research Institute, 20014 San Sebastian, Spain; daniela.gerovska@biodonostia.org; 4 IKERBASQUE, Basque Foundation for Science, Calle María Díaz Harokoa 3, 48013 Bilbao, Spain; 5 Department of Pathology, Onkologikoa Foundation, 20014 San Sebastián, Spain; rrezola77@gmail.com; 6 Wallenberg Center for Molecular Medicine (WCMM), 58185 Linköping, Sweden; alien.balian@liu.se; 7 Department of Physics, Chemistry and Biology, Linköping University, 58185 Linköping, Sweden; 8 Department of Oncology, Onkologikoa Foundation, 20014 San Sebastián, Spain; ozuriarraiz@gmail.com (O.Z.B.); lidelar@hotmail.com (L.L.G.); anderu@onkologikoa.org (A.U.)

**Keywords:** breast cancer, nucleic acid probes, cancer diagnostics, nuclease activity, biomarkers

## Abstract

**Simple Summary:**

A diagnostic biomarker for the detection of breast cancer remains an unmet clinical need despite decades of intensive research efforts. Herein, we describe, for the first time, the use of nuclease activity as a biomarker to discriminate between healthy and cancer biopsy samples. We have identified a panel of three nucleic acid probes able to target nucleases derived from breast cancer tumors with high sensitivity and specificity. These results are in good agreement with histopathological analysis as the diagnostic gold standard. Moreover, these findings support nuclease activity as a potential adjacent diagnostic tool and shed light on the use of nuclease activity as a detection biomarker in breast cancer.

**Abstract:**

Breast cancer is one of the most common pathologies diagnosed in the clinical practice. Despite major advancements in diagnostic approaches, there is no widely accepted biomarker in the clinical practice that can diagnose breast malignancy. Confirmatory diagnosis still relies on the pathological assessment of tissue biopsies by expert pathologists. Thus, there is an unmet need for new types of biomarkers and novel platform technologies that can be easily and robustly integrated into the clinic and that can assist pathologists. Herein, we show that nuclease activity associated to malignant tumors can be used as a novel biomarker in breast cancer, which can be detected via specific degradation of nucleic acid probes. In this study we have identified a set of three chemically modified nucleic acid probes that can diagnose malignancy in biopsy samples with high accuracy (89%), sensitivity (82%) and specificity (94%). This work represents a breakthrough for the potential clinical use of nuclease activity as biomarker, which can be detected via nucleic acids probes, for the clinical diagnosis of malignancy in breast tissue biopsies. This platform technology could be readily implemented into the clinic as adjunct to histopathological diagnostic.

## 1. Introduction

Breast cancer is one of the most frequently diagnosed malignancies and the most common cause of cancer death in women all over the globe. In the last two decades, the clinical management of breast cancer patients has been greatly facilitated by the availability of several biomarkers with prognostic and predictive values, such as human epidermal growth factor receptor 2 (HER2/neu) gene amplification, elevated levels of estrogen receptor (ERs) or progesterone receptor (PR) and proliferation-related genes, such as the proliferation index (Ki67), that helped stratify patients for receiving appropriate therapy and predict the likelihood of therapy response [1]. These biomarkers are the most well-established in clinical use, and their expression status in tumors is routinely evaluated [2]. Importantly, the status of ER, PR, HER2 and Ki67 has been correlated with the well-established five molecular subtypes of breast cancer: luminal A, luminal B, HER-2-enriched, basal like also referred to as triple negative breast cancer (TNBC) and normal like. Luminal A tumors express ER but not HER2 and have low Ki-67, while Luminal B tumors express less ER-related genes but have elevated proliferative index and may exhibit HER2 overexpression. HER2-enriched tumors are more aggressive compared with the luminal tumors that are indolent in nature. This subtype is negative for ER and PR expression and the HER2/neu is overexpressed in these tumors. TNBCs have acquired their name due to negative expression of ER, PR and HER2. Poor prognosis and high recurrence rate have been correlated with this subtype [2,3]. While clinical implementation of these markers significantly improved overall survival and decreased recurrence of disease, the expression of ER and PR can still be heterogeneous, and proliferative status (Ki67) can vary widely within a single tumor [4]. Moreover, inherent errors with biopsy sampling, size of the available specimen for further marker staining and margins assessment after surgical resection can be expected. Thus, a pathological diagnostic greatly depends on the quality of the sample, preparation of histological sections and careful clinical and pathological correlation [5]. Despite these limitations, histopathology is still considered the gold standard for the analysis of breast cancer biopsy samples [6].

In the last decade, several emerged biomarkers, such as serum carcino-embryonic antigen (CEA) [7], carbohydrate antigens (CA) 13-5, CA27-29 [8], *BRCA1/2* genes [9] circulating tumor cells (CTCs) [10], tumor infiltrating lymphocytes (TILs), etc., have shown to be useful in prognosis, monitoring treatment response, prediction of progression and survival. Despite all efforts, there is still a need for novel biomarkers that can be translated in the clinical practice, especially for the early detection of breast cancer [11,12]. The implementation of many other promising candidates awaits thorough clinical validation and standardization. The sample of choice (e.g., blood, tissue and urine), sample size and methods of preparation directly influence the biomarker performance [13,14,15]. Ultimately, a useful biomarker that can revolutionize the current standard of care should be able to provide a global overview of the tumor status, in a simple and efficient manner. Herein, we propose nuclease activity as a functional biomarker in cancer, most specifically for breast cancer tumors.

Among proteins with catalytic activity, nucleases are enzymes that degrade nucleic acids by hydrolyzing the phosphodiester bonds that join the sugar residues [16]. They are critical components of the biological processes [17,18,19] and have been previously acknowledged as possible tumor markers [20,21,22,23,24,25]. In cancer cells, expression of nucleases has been reported, both at the gene and protein levels [26,27]. The expression levels of DNases have been linked to carcinogenesis, progression and prognosis of cancer [28,29,30,31,32]. Furthermore, RNases have been found to have altered expression in cancer cells [33,34,35], and, also, in the blood of cancer patients [36], they have been found to be either elevated [37,38] or decreased [39] in serum. These studies went further, to propose the altered nuclease activity in cancer as possible biomarker of disease. However, the lack of a standardized methodology and the absence of a robust platform of investigating nuclease activity tempered somehow the enthusiasm of pursuing nucleases as valid biomarkers in cancer. 

The availability of several chemically modified nucleotides [40] offers the possibility of tailoring oligonucleotide substrates for targeting nuclease activity derived from cancer, in a specific and sensitive manner [41]. Therefore, we hypothesized that the wide diversity of nucleases, together with their altered expression and deregulated activity in cancer, can be exploited as a new type of biomarker for the diagnosis, prognosis and possibly prediction of treatment response in many cancers, by using short and specific nucleic acids substrates. Moreover, once the specific substrate sequences are identified, they can be incorporated into various detection approaches, such as magnetic resonance imaging (MRI), fluorescence, colorimetric or electrochemical methods. One major advantage of this technology is that the enzymatic activity of nucleases can act as an intrinsic signal amplification module, where each substrate degradation event results in signal accumulation, therefore eliminating the need for the PCR amplification step required in most of the nucleic acids–detection systems. This feature is desirable for the development of early and sensitive detection methods.

We have previously demonstrated that we can differentiate healthy cells from cancer cells by detecting their associated nuclease activity, using chemically modified nucleic acid probes as substrates [42]. Specifically, we have identified the presence of a specific DNase activity profile associated with breast cancer cells (SKBR3). Therefore, we postulated that nuclease activity associated to cancer cells (in cultures) can be used as a novel biomarker in cancer, and more specifically in breast cancer. Interestingly, in another study CTCs enriched from the blood of patients with stage IV breast cancer could be detected via their intracellular nuclease activity. However, this study only addresses the detection of advanced breast cancer, when the possibility for patient´s recovery is very low and this approach is not suitable for screening of general population [43]. However, specific nuclease activity derived from human tumors has not been reported yet, and this could provide valuable clinical information for early stage disease, when several therapeutic options could provide a better outcome for the patient. 

We hypothesized that we can use nuclease activity to identify breast cancer tumors. We focused our efforts in developing nucleic acid substrates or probes that can specifically detect nuclease activity derived from breast tissue biopsies. We designed and performed a proof-of-concept study for the screening and identification of nuclease activity as biomarker in breast cancer tumors. In this study, we used computational analysis to identify the best performing nucleic acid probes. Thus, we report on the identification of a panel of three nucleic acids probes that can correctly diagnose 54 out of 61 patients (89% accuracy) with high sensitivity (82%) and specificity (94%). To the best of our knowledge, this is the first report on the use of chemically modified nucleic acids probes for the detection of malignancy in breast tissue biopsies via a specific tumor-associated nuclease activity.

## 2. Results

### 2.1. Retrospective Study

**Figure 1:**
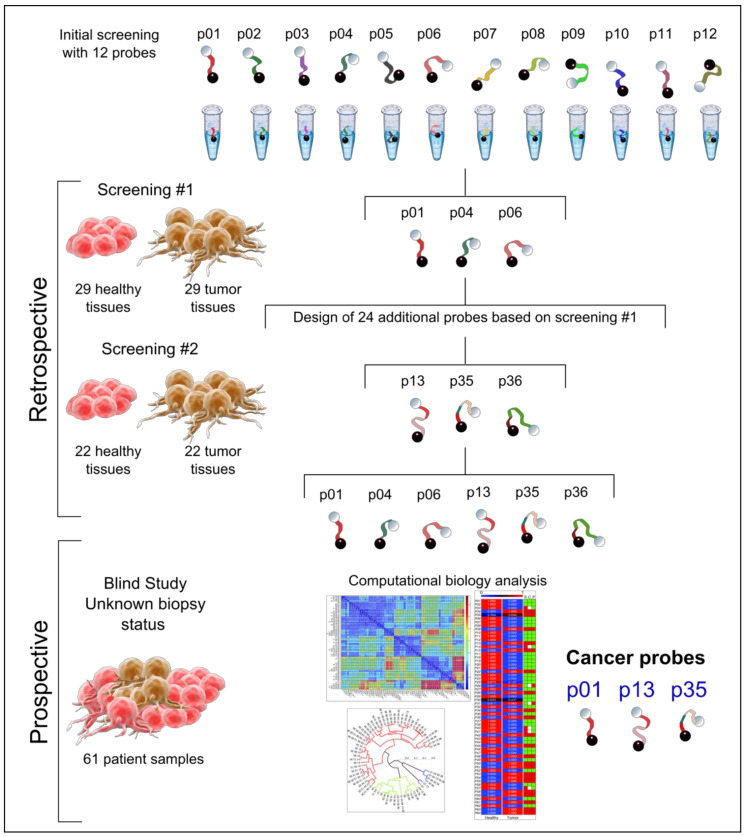
Study workflow: nuclease activity screening and computational biology analysis.

We sought to screen the profile of nuclease activity derived from healthy and breast-tumor tissues. Nuclease activity of a total of 102 samples from 51 patients (paired samples) was evaluated by a two-step screening method. Clinical information for all patients in this study (retrospective and prospective cohorts) is provided in Table 1. Figure 1 depicts the study workflow. The nucleic acid probes library design and the selection of probes for each screening round are specified in the Materials and Methods section.

#### 2.1.1. First-Step Retrospective Screening for Detecting the Blueprint of Nuclease Activity in Tumors

**Figure 2:**
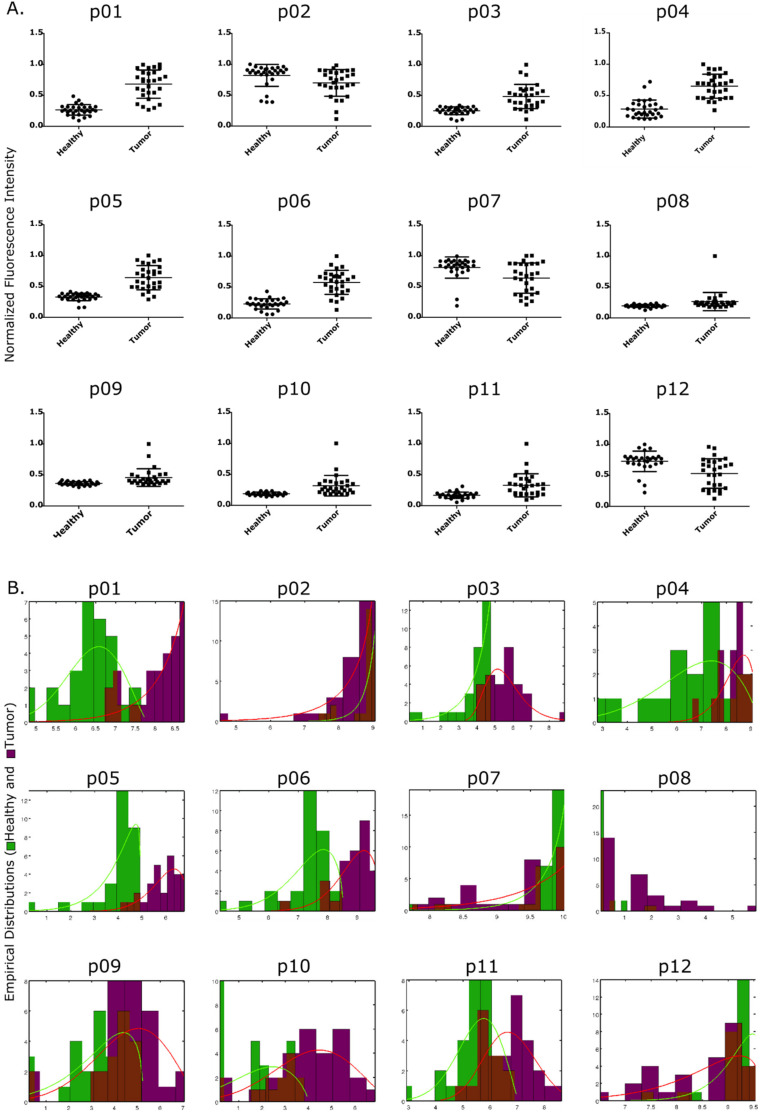
First step retrospective study: (**A**) Performance of the 12 generic probes (p) in differentiating between the healthy and tumor groups for the 29 paired samples (58 total). The plots represent the average and standard deviation of the normalized fluorescence intensity, showing the degradation of each individual probe. (**B**) Histograms and empirical distribution functions of the probes for healthy (**green**) and tumor (**red**) samples for the identification of the best performing probes. The empirical distribution functions are represented by the continuous lines in green and red for the healthy and tumor samples, respectively. Less overlap between distributions corresponds to better discrimination between the healthy and tumor samples.

We screened the 12 generic probes (p01–p12) against 58 paired tissue samples (29 healthy breast tissues and 29 tumor breast tissues) obtained from 29 patients. Detailed probe sequences are included in Table S1. The screening of these paired tissue samples showed the ability of the DNA-based probes to discriminate between tumor and healthy counterparts. These results confirm the existence of a differential nuclease activity profile associated to tumor versus healthy tissues. Representative examples for the detection of specific nuclease activity in these samples are shown in Figure S1. Herein, the increase in fluorescence intensity represents the increase in nuclease activity as result of the probe degradation event for each sample. In this initial screening, we identified the presence of a potent DNase activity associated with tumor tissues across all patient samples that could efficiently degrade 5 of the 12 probes, namely DNA (p01), All 2’-Fluoro (p03), Pyr 2’-Fluoro DNA (p04), Pyr 2’-Fluoro RNA and Pur 2’-Fluoro DNA (p06) (Figure 2A). These results are in good agreement with our previous findings in breast cancer cells, where we showed that the DNA substrates are better digested by breast cancer cells than the RNA substrates, under the same conditions [42]. Additional analysis of the discrimination capability of each of the 12 probes was performed by computational analysis, determining the probability of each probe to be degraded by healthy and tumor tissue samples. This was accomplished by calculating the overlap of probability distribution functions for the healthy and tumor samples, as detailed in the Materials and Methods section. The probes with the best discrimination capabilities are those with smaller overlap between healthy and tumor probability distribution functions (Figure 2B). In agreement with the experimental data, this analysis revealed the preference of the nucleases for the DNA-containing substrates, as shown by the degradation profile and the computational prediction of probes, such as the natural DNA (p01) and its chemically modified derivatives, Pyr 2’-F DNA (p04) and Pur 2’-F DNA (p06) probes, when compared to the RNA based probes. Therefore, we have selected p01, p04 and p06 probes and analyzed their combined ability to predict the tissue status (healthy or tumor) of the 29 patient samples. These probes have better predicted probability of discriminating the healthy tissues (Figure S2A–C) from the tumor tissue (Figure S2D–F).

#### 2.1.2. Second-Step Retrospective Screening, Using Tailored Probes for Targeting Tumor Nucleases

Forty-four retrospectively collected paired tissue samples (22 healthy and 22 tumor breast tissues) were screened for the nuclease activity, using 24 probes designed as described in the Materials and Methods section. To identify which probes can better discriminate between healthy and tumor tissues, we again performed computational analysis on the raw fluorescent data from the nuclease activity assay and predicted the overlap of the healthy and tumor probability distribution functions (Figure S3). We identified 3 new probes, namely poly A (p13), AAACCC chimera (p35) and AAAUUU chimera (p36), that were further analyzed for their probability to predict tissue status, either healthy or tumor (Figure S4). This time, their combination was able to predict more accurately the identity of the tumor samples.

### 2.2. Prospective Study

To prove the efficiency of our approach based on nuclease activity, we designed a prospective study, using fresh biopsy samples derived from 61 patients. Clinical information for all patients in the prospective cohort is provided in Table 1. The samples were tested for nuclease activity, as previously described, by using the set of six probes reported in the retrospective study. The samples were tested without a priori knowledge of their clinical status (healthy or tumor), with the idea of conducting a blind study. To report the most accurate probability of healthy or tumor status for each sample, we performed two computational analyses. First, we conducted a pre-analysis with the prospective samples to identify the best-performing combination of probes, and then we used this combination of probes to classify each sample as either being healthy or tumor. A detailed description of each analysis is provided below.

### 2.3. Pre-Analysis to Identify the Best Performing Probes for Tumor Diagnosis

We performed a pre-analysis by using the six probes identified in the first-step retrospective screening (p01, p04 and p06) and second-step retrospective screening (p13, p35 and p36). We searched, from all possible 63 combinations of these six probes (predictors), for the optimal combination of probes that could discriminate healthy from tumor tissues in the prospective study. We preprocessed the measurements of the six probes, as in the retrospective case. Next, we estimated the probability of a prospective sample to be classified as healthy or tumor with all the possible combinations of predictors. While searching for clusters of combinations of predictors, we observed, in the map of distances of prediction probabilities (Figure S5A), that a group of 14 combinations of probes (marked with an ellipse) shows closer distances with all the other combination of probes. These are the same 14 combinations of probes forming a branch in the hierarchical clustering in Figure S5B and in the cluster in the Principal Component Analysis (Figure S5C). Therefore, we deduce that these 14 combinations have the shared predictive potential of all the 63 combinations. Interestingly, these 14 combinations contain probes from the two retrospective screenings. We analyzed the probabilities of each patient sample to be predicted as healthy or as tumor in the heat map in Figure S5D, and when we compared them to the real state of each sample (presented in the column to the right of the figure as “S”), we found a good predictive performance and suitability of all the possible combinations of the six predictors for the discriminant analysis between healthy and tumor tissues. 

**Figure 3:**
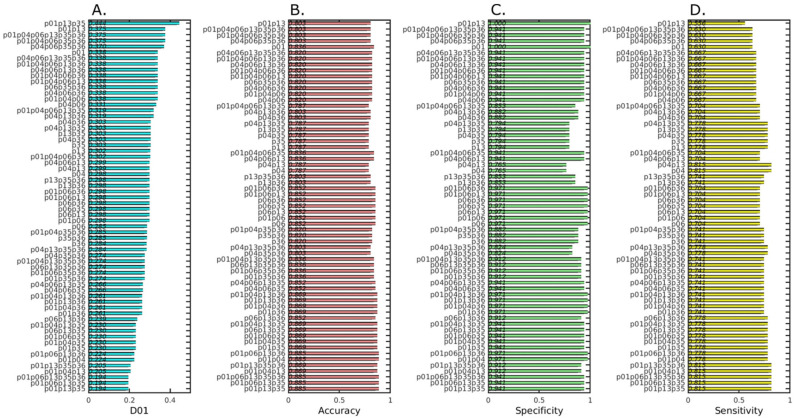
Bar plots of the quality measurements associated with the Receiver Operating Characteristic (ROC) of the performance of the predictions achieved with each of the combinations of predictor variables for the discriminant analysis between Healthy and Tumor on the prospective dataset. (**A**) D01, distance to the optimal point (0, 1) of the ROC space. (**B**) Accuracy. (**C**) Specificity. (**D**) Sensitivity. The bars in all bar plots are sorted in descending distance to the optimal point (0, 1) of the ROC space.

Finally, to find which members of the clusters reported in Figure 3 have the best prediction capabilities, we calculated the performance of all the possible combinations of the six selected probes, using performance metrics such as Receiver Operating Characteristic, where we selected the distance to the optimal point (0, 1) of the Receiver Operating Characteristic (ROC) space (D01, Figure 3A), the accuracy (Figure 3B), specificity (Figure 3C) and sensitivity (Figure 3D). Based on these characteristics, we observed that several combinations of probes: (p01, p06, p13, p35 and p36), (p01, p06, p13 and p35) and (p01, p13 and p35) achieved the same results for the best ROC parameters: D01 (0,194), accuracy (0.885), specificity (0.941) and sensitivity (0.815). Out of the three best-performing combinations, we chose the combination with the least number of probes (p01, p13 and p35) that reported the minimal distance to the optimal point (0, 1) of the ROC space (Figure 3A at the bottom), and we named them “cancer probes”.

### 2.4. Analysis and Prediction of the Clinical Status of Each Prospective Sample Using the Three Cancer Probes

**Figure 4:**
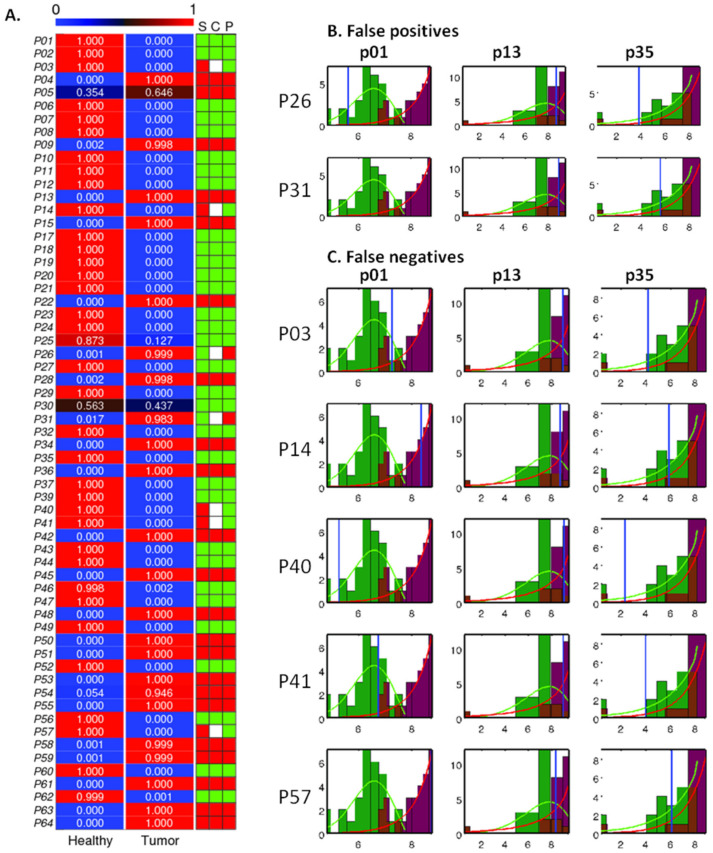
Predictions of the best combination of probes (p1, p13 and p35) on the prospective dataset of 61 patients. (**A**) Heatmap of the probabilities of each sample to be predicted as healthy or tumor. The table to the right marks: S, real status of the sample; P, prediction of the status; and C, the comparison between them. Healthy in green, tumor in red, and mismatch between real status and prediction in white. (**B**,**C**) Histograms and empirical distribution functions of the probes in healthy (green) and tumor (red) samples of the false positive (**B**) patient samples (P26 and P31) and false negative (**C**) patient samples (P03, P14, P40, P41 and P57). The empirical distribution functions are represented by the continuous lines in green and red for healthy and tumor samples, respectively. The blue vertical line marks the decision boundary of the discriminant analysis. Less overlap between distributions corresponds to better discrimination between healthy and tumor samples.

The three probes identified by computational analysis (p01, p13 and p35) (Figure 3) were used to classify the patient tissue biopsies as healthy or tumor. As indicated in the Materials and Methods section, 3 of the 64 samples (derived from patients 16, 33 and 38) were not tested, due to low amount of tissue available for processing. Thus, 34 samples were predicted as being healthy and 27 as being tumor, for a total of 61 samples. Figure 4 shows the prediction of samples using the three selected cancer probes. The prediction provides a score for each sample, from 0 to 1, and the decision of healthy or tumor was obtained. Next, the predicted diagnosis (P) and the real clinical diagnosis (S) are compared (C), as indicated in the right column in Figure 4A. The color code in this column indicates healthy samples in green, tumor samples in red and, in white, the disagreement between S and P. Uniformity in color for S, C and P indicates agreement in diagnosis for pathology and cancer probes. When compared to the pathology results, our panel of probes was able to detect cancer in 22 out of 27 malignant lesions and correctly identified 32 out of 34 non-malignant lesions as negative for cancer. The histograms and empirical distribution of the false negatives and false positives reported in this study are shown in Figure 4B. To complete this analysis, representative H&E pictures of healthy and tumors samples, along with the false positives and false negatives samples predictions, are shown in Figure S6. The histopathological diagnoses of the 61 patient samples included in this study are listed in Table S2, where different type of malignant lesions were observed, such as invasive ductal carcinoma (IDC), ductal carcinomas in situ (DCIS) with IDC, one mucinous carcinoma and two papillary carcinomas. Of the 34 non-malignant lesions, 18 were found to be normal benign, and the rest presented different atypical lesions. In this study, we have obtained a positive predictive value of 92% and the negative predictive value of 87%. Detailed calculations of these values are presented in Table S3. 

### 2.5. Serum Stability of the Three Cancer Probes

To verify the suitability for clinical use, the three cancer probes identified in this study (p01, p13 and p35) were further tested in human serum from a healthy donor. Nuclease assay was performed on the serum sample, along with healthy and tumor tissue homogenate samples. Figure S6 shows that the selected probes are resistant to endogenous nucleases present in human serum, while being highly susceptible to degradation by tumor tissue but not by healthy tissue. As expected, we observed high resistance for the probes p13 and p35, giving the presence of 2’-Fluro and 2’-O-Methyl modifications of the nucleic acids in both probe sequences (see Table S1 for details). The native DNA sequence of the p01 probe has shown slightly higher degradation compared to the modified probes. This behavior is expected for an unmodified DNA probe in the presence of serum endogenous nucleases. With this in mind, the cancer probes p13 and p35 would be more suitable for in vivo studies and potential clinical translation.

## 3. Discussion

We have previously proposed nuclease activity as a novel biomarker for cancer using in vitro cell cultures [36]. Herein, we go one step further with a proof-of-concept study, using biopsy samples. Moreover, in this study, we showed the possibility of using the nuclease activity as a valid biomarker for discriminating healthy from malignant breast tissue biopsies. 

Given the high cell heterogeneity of the tumor environment and the documented presence of altered nuclease expression in cancer, we hypothesized that there exists a global and differential nuclease activity associated to tumors versus healthy tissues. To test our hypothesis, we designed a library of nucleic acids probes (substrates), to screen for a specific nuclease activity associated to tumors but not to healthy tissues. Since endogenous nucleases rapidly digest natural nucleic acids, such as DNA and RNA, we designed these nucleic acids probes with various chemical modifications, to increase their resistance towards non-specific nucleases. Moreover, we combined various sequences and chemical modifications, to increase the stringency of the screening process. We have identified DNases as the main type of nucleases with high activity in breast tumor tissues, based on the efficient degradation observed for the DNA-based probes. These findings are in very good agreement with previous work in our group on breast cancer cell lines [36]. After two rounds of screening using a total of 36 probes, we have identified three probes that, in combination, can efficiently detect breast malignancy with an accuracy of 89%. These results are noteworthy, showing that human tumors can be differentiated from normal tissues by using chemically modified nucleic acid probes and nuclease activity as a biomarker.

We found two false positives and five false negatives by using our probe combination approach. The histopathological assessment of these seven samples (H&E staining) is shown in Figure S7. For the two false positives, we suspect a pre-diagnosis scenario, where these three probes can detect early malignant transformation, at the molecular level, before any phenotypical changes occurred. In one of the cases, the patient was initially macro diagnosed, at receiving, with malignant (in situ) inflammatory breast cancer. The diagnosis was then changed to a post-surgical granulomatous reaction based on the histopathological results (patient 31 in Figure S7C, upper panels) conducted by an expert pathologist. Another patient was also diagnosed with a granulomatous reaction based on a silicon implant (patient 26). Additionally, errors with biopsy sampling and, in some cases, quality of the tissue available for the nuclease activity assay could also explain the discordance between our results and the pathologist’s conclusions for these two cases. The detection of tissue nuclease activity with chemically modified probes could provide additional information reflecting a global status, at the molecular level and in a shorter period of time. In this context, we envision that this technology can be useful for the intraoperative evaluation of the sentinel lymph node biopsy and the marginal status.

This approach could help pathologists to reach the best conclusions and provide the right diagnosis for the borderline cases. This is very relevant especially in those situations when misdiagnosis could lead to over-treatment or under-treatment of the patients.

## 4. Materials and Methods

### 4.1. Study Design

In this study, we developed a workflow (Figure 1) to identify nuclease activity associated to breast tissue biopsies. This study was carried out in two phases: (i) a two-step screening retrospective study, using paired frozen tissue biopsy samples. Both samples, tumor and tumor surrounding healthy tissues, were obtained from the same breast quadrant of the patient “paired samples”, allowing a margin of several centimeters. Next, the tissues were examined by an expert pathologist, to confirm the nature of the sample. The samples were collected by the Biobank of the Basque Country (2014–2016) and stored at −80 °C. (ii) A prospective study of patient tissue biopsies freshly collected by the Hospital Onkologikoa Foundation (2016–2019) was also carried out. This study was approved by the ethical committee of the University Hospital Donostia (San Sebastian-Donostia, Spain), with the clinical protocol number FJH-SDC-2016-01. Patients scheduled for biopsy from the national screening program of breast cancer and patients suspected of breast cancer were enrolled in the study. All patients provided written informed consent to be admitted in the study. The standard diagnosis workflow [44] used in this study followed the recommendation of the European Guidelines for Quality Assurance in breast cancer screening and diagnosis [45]. All the experiments were conducted according to the principles expressed in the Declaration of Helsinki.

#### 4.1.1. Retrospective Studies

The retrospective screening was divided in two steps: (a) an initial screening, using the 12 general probes, to identify the degradation profile or blueprint of the tumor associated nucleases; and (b) a follow-up screening, using a new set of probes, designed based on the information obtained in the initial screening. Thus, the second screening was intended to target tumor nucleases in a more specific manner. The size of the retrospective study was not pre-specified. 

##### Tissue Preparation for the Retrospective Study

A total of 102 frozen tissues (paired, healthy and tumor) from 51 patients, stored in 2 mL vials, at −80 °C, were obtained from the Biobank of the Basque Country, Spain. The tissues were thawed over ice and weighted. The buffer for tissue homogenization (HB) was prepared by adding a protease inhibitor cocktail (10 µL/mL) to PBS containing Ca2+ and Mg2+. Tissues were then homogenized in HB to a final concentration of 0.5 g/mL. Next, the homogenates were centrifuged at 14,000 rcf, for 15 min, at 4 °C. The supernatants were then collected in a fresh 1.5 mL Eppendorf tube and were either kept on ice for immediate nuclease assay experiments or stored at −80 °C for further use.

#### 4.1.2. Prospective Study

Patients were enrolled in this study after signing a written informed-consent form. The size, N, of the prospective study was calculated by using normal approximation in Z statistics [46,47], using the following equations:

N = AB/(E/S)^2^ (1)

where

A = (1/q_1_ + 1/q_0_) (2)

B = (Zα + Zβ)^2^ (3)

E/S is the standardized effect size; for an effect size E = 0.55 and assuming that the one standard deviation of the outcome is S = 1, E/S = 0.55. Since the proportion of subjects in Group 0 (healthy) is q_0_ = 0.58, the proportion of subjects in Group 1 (tumor) is q_1_ = 1 − q0 = 0.42. Thus, using equation (2), A = 4.105. By imposing a threshold for the probability for rejecting the null hypothesis, α = 0.2, and another threshold for the probability of failing to reject the null hypothesis under the alternative hypothesis, β = 0.2, we obtained, using the two-tailed Z statistics, the standard normal deviates for α and β, Zα = 1.282 and Zβ = 0.842, respectively. Thus, using equation (3), B = 4.508. Finally, by applying equation (1), we determined that the total group size N is 61. After sample collection, 3 of the 64 samples (derived from patients 16, 33 and 38) were not tested, due to the low amount of tissue available for processing. Thus, these patients’ samples were excluded from this study, and we analyzed a total of 61 samples.

##### Tissue Preparation for the Prospective Study

Breast tissues from 61 patients were collected during surgical biopsy or breast surgery. Tissues were stored on ice and processed within 1 hour, to preserve as much as possible the integrity of the nucleases. Typically, the biopsy pieces weighted between 0.060 and 0.150 g. Tissues were washed in cold PBS and homogenized in homogenization buffer (HB), using a handheld homogenizer (Bio-Gen Pro200 from PRO Scientific, CT, USA) in round-bottom 2 mL tubes. Samples were kept on ice for the entire period of processing, and the resulting supernatants were then collected in a fresh 1.5 mL Eppendorf tube, as previously indicated for the retrospective study. Subsequently, the nuclease activity assay was performed.

### 4.2. Probe Library Design

The initial library of 12 probes was designed to cover a broad spectrum of known or suspected types of nuclease activities (e.g., DNase) that are present in tumors during the malignant transformation. Thus, this probe library comprises sequences containing natural nucleic acids, DNA and RNA, as well as sequences containing nucleic acids fully modified with 2’-Fluoro and 2’-O-Methyl chemistries. These 4 probes were named according to the nature of their nucleic acid makeup, as follows: DNA probe, RNA probe, All-2´-Fluoro probe and All-2´-O-Methyl probe. To simplify the nomenclature for the data analysis, all probes were also given a probe number, from p01 to p12 (Table S2). We also combined chemically modified (2’-Fluoro or 2’-O-Methyl) purines or pyrimidines with natural purines or pyrimidines of DNA or RNA for each case. As a result, 8 additional sequences were obtained: Pyr 2’-Fluoro DNA, Pyr- 2’-Fluoro RNA, Pur-2’-Fluoro DNA, Pur-2’-Fluoro RNA, Pyr 2’-O-Methyl DNA, Pyr- 2’- O-Methyl RNA, Pur-2’- O-Methyl DNA and Pur-2’- O-Methyl RNA. Detailed information about all sequences is provided in Table S2. To further increase the specificity of the probes for tumor nucleases, we planned a second round of screening (second retrospective screening) by designing additional nucleic acid probes based on the 3 probes that have shown the best degradability profile for breast tumor tissues, namely the DNA probe (p01) and the 2’-Fluoro DNA containing probes (p04 and p06), while having the 2’-O-Methyl as the resistant moiety. We therefore designed 24 additional probes (p13 to p36) (Table S2), containing 2’-Fluoro modified polymeric sequences (poly A, poly C and poly U) and chimeric sequences, obtained from a combination of 2’-Fluoro and 2’-O-Methyl modified nucleotides. These 24 chimeric probes were tested in a second cohort of 44 retrospectively collected tissues biopsies, with 22 healthy breast tissues and 22 tumor breast tissues collected from 22 patients (paired samples). By introducing various chemical modifications at selected positions in the probe sequences, we achieved a two-fold degree of specificity: (a) We minimized interferences from non-specific nucleases (typically represented by DNase1 and RNases) that have a high propensity of cleaving natural substrates; (b) we increased the stringency of the selection, to favor those nucleases with altered activity in tumors.

### 4.3. Probes Synthesis

Oligonucleotide probes were synthesized and purified by Biomers.net (Ulm, Germany), as we previously described [48]. Briefly, all the probes were synthesized with the fluorescein amidite (FAM) fluorophore at the 5’-end and the quencher tide2 (TQ2) at the 3’-end. Thus, the initial fluorescence of the probes was effectively quenched via fluorescence resonance energy transfer (FRET). Synthesis was performed by using standard solid-phase phosphoramidite chemistry, followed by high-performance liquid chromatography (HPLC) purification. The probe identities were confirmed with matrix-assisted laser desorption ionization–mass spectrometry (MALDI–MS). The purity of the probes, as assessed with HPLC analysis, was typically greater than 95%.

### 4.4. Nuclease Activity Assay

Nuclease activity assay was performed as previously reported [49], with small modifications. Specifically, for each reaction, 5 μL of sample (HB or samples homogenates) was combined with 4 µL PBS (+/+) and 1 μL (50 pmoles) of oligonucleotide probe (nuclease substrate) and incubated at 37 °C, for 1 hour. After the incubation period, the reaction was stopped by adding 295 μL of PBS (-/-) supplemented with 10 mM EDTA. Next, 95 μL of each sample was loaded in triplicates into 96-well plates (96F non-treated black microwell plate, Thermo Fisher Scientific). Fluorescence intensity was measured with a fluorescence microplate reader (Synergy HT, BioTek, Winooski, VT, USA), using the filter settings for FAM (excitation/emission (ex/em), 494/521 nm)).

### 4.5. Computational Biology Analysis

The retrospective measurements were used as a training set to estimate the heuristic probability distribution functions of healthy and tumor conditions used to select the best probes. To correct the background signal, during the preprocessing stage for each patient, we subtracted the buffer control probe measurement from the healthy- and tumor-sample probe measurements. To equalize the corrected signal, we applied a log2 transform after setting to 1 the corrected values lower than 1. To analyze the clustering of combinations of predictors, we used Principal Component Analysis (PCA), the map of distances of probabilities between combinations of predictors based on the 1 minus the Pearson correlation (ρP) metric, MρP, and the hierarchical clustering using the MρP metric and the unweighted average distance (UPGMA) (also known as group average) linkage method. The heuristic probability distribution functions of healthy and tumor conditions were predicted by fitting the corresponding log2 transformed signals, using the generalized extreme value model.

To estimate the discrimination capability of each probe, we calculated the overlap of the healthy and tumor probability distribution functions. The probes with best discrimination capabilities are those with smaller overlap between healthy and tumor probability distribution functions. 

To estimate the healthy and tumor probabilities of the samples from the prospective studies, first we preprocessed the measurements of the probes, as in the retrospective case. Next, we selected the set of best predictors from the two screenings of the retrospective study and we calculated the performance of all the possible combinations of selected probes, using performance metrics of the Receiver Operating Characteristic (ROC), namely the distance to the optimal point (0, 1) of the ROC space (D01), the accuracy and the specificity. Finally, we chose the combination of probes with the minimal distance to the optimal point (0, 1) of the ROC space. Data processing and graphics were performed with in-house developed functions in Matlab (MathWorksTM, Natick, MA, USA).

### 4.6. Histopathological Methods 

Fresh surgical biopsy tissues were randomly fragmented into smaller pieces. Fragments with a surface area of 5–8 × 4–6 mm and with a thickness of approximately 4 mm were divided into 2 halves. One half was fixed in 4% formaldehyde, for further histopathological examination, and the other half was immediately transported in ice, for nuclease activity assessment in the prospective screening. The fixed tissues were embedded in paraffin overnight, and sections of 3 or 4 microns were cut and mounted on slides, followed by staining with Hematoxylin–Eosin (H&E). All samples slides were imaged with a digital light microscope for standard histopathological analysis. The process was carried out blindly, without knowledge of the nuclease activity assay results.

## 5. Conclusions

In summary, we reported on a panel of three probes that have the capability to differentiate between breast cancer tumors and normal healthy tissues, with an accuracy of 89%, sensitivity of 82% and high specificity of 94%. Moreover, the detection of nuclease activity derived from breast cancer tissues offers a robust and easy platform that could readily be implemented in the clinic as an adjunct method to the standard histopathological interpretation of tissue biopsies. As a future direction, nucleic acids–based probes could open the doors for the development of alternative and more efficient diagnostic approaches for other types of cancer and even other diseases. We envision that, once a specific set of probes is identified, the sequences can be incorporated into MRI probes that can work as contrast agents, thus adding diagnostic value to the non-invasive detection of cancer through the use of MRI machines, which are widely implemented into the clinic. We and others are currently exploring the translation of enzymatic-based probes into MRI probes for diagnostic purposes [50,51,52,53]. Furthermore, we are currently exploring the translation of oligonucleotide sequences into the MRI probes for diagnostic purposes.

## Figures and Tables

**Table 1:** Patient clinical information.

	Retrospective	Prospective
	*n* = 51	*n* = 61
Age (years)		
Mean	62	56
Median (range)	63 (36–85)	52 (40–77)
Clinical tumor size		
<20 mm	34 (66.7%)	
>20 mm and ≤50 mm	12 (23.5%)	
> 50 mm	5 (9.8%)	
Unknown	0	
Grading		
G1	0	8(29.6%)
G2	12 (23.5%)	14 (51.9%)
G3	32 (62.7%)	2 (7.4%)
Unknonwn	7 (13.7%)	3 (11.1%)
ER/PR status		
Both negative	24 (47.1%)	4 (14.8%)
One or both positives	27 (52.9%)	15 (55.6%)
Unknown	0	8 (29.6%)
HER2 status		
Negative	40 (78.4%)	13 (48.1%)
Positive	11 (21.6%)	6 (22.2%)
Unknown	0	8 (29.6%)
Lymphovascular invasion		
No	46 (90.2%)	15 (55.6%)
Yes	5 (9.8%)	7 (25.9%)
Unknown	0	5 (18.5%)
Histological tumor type		
Ductal invasive	38 (74.5%)	21 (34.4%)
Lobular invasive	3 (5.9%)	1 (1.6%)
Other	7 (13.7%)	5 (8.2%)
Unknown	3 (5.9%)	0
Benign	NA	34 (55.7%)

## Data Availability

The data presented in this study are available in the manuscript and in the Supplementary Materials. Additional raw data are available on request from the corresponding author.

## Supplementary Materials

The following are available online at https://www.mdpi.com/2072-6694/13/2/276/s1. Table S1: List of oligonucleotide probes used in this study, Table S2: Patient histopathological diagnostic data, Table S3: Contingency table of the discriminant analysis, using the best combination of probes (p01, p13 and p35), Figure S1: Representative raw data for the screening of nuclease activity in breast tissue biopsies, Figure S2: Predictions with the combination of probes (p01, p04 and p06) of the healthy and tumor samples from Retrospective Screening #1, Figure S3: Empirical distribution functions of the 24 additional probes on Retrospective Screening #2, Figure S4: Predictions with the combination of probes (p13, p35 and p36) of the healthy and tumor samples from Retrospective Screening #2, Figure S5: Search of the optimal combination of the six (p01, p04, p06, p13, p35 and p36) predictor variables for the discrimination analysis between healthy and tumor on the prospective dataset, Figure S6: Stability in serum of the cancer probes, Figure S7: Hematoxylin and Eosin (H&E) staining of representative samples of breast tissues.
