# Supplementary material for: Discovery and Proof-of-Concept Study of Nuclease Activity as a Novel Biomarker for Breast Cancer Tumors
Source: Cancers (Basel). 2021 Jan 13;13(2):276. doi: 10.3390/cancers13020276 (PMC7828568; doi:10.3390/cancers13020276)
Supplement: Supplementary file 1 [file cancers-13-00276-s001.pdf]

cancers

Supplementary Materials: Discovery and Proof-of-Concept Study of Nuclease Activity as a Novel Biomarker for Breast Cancer Tumors

Luiza I. Hernandez, Marcos J. Araúzo-Bravo, Daniela Gerovska, Ricardo Rezola Solaun, Isabel Machado, Alien Balian, Juliana Botero, Tania Jiménez, Olaia Zuriarrain Bergara, Lide Larburu Gurruchaga, Ander Urruticoechea and Frank J. Hernandez

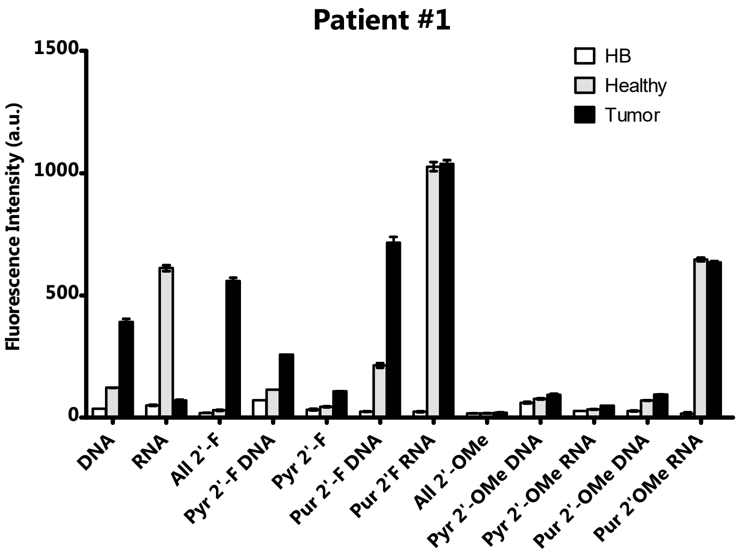

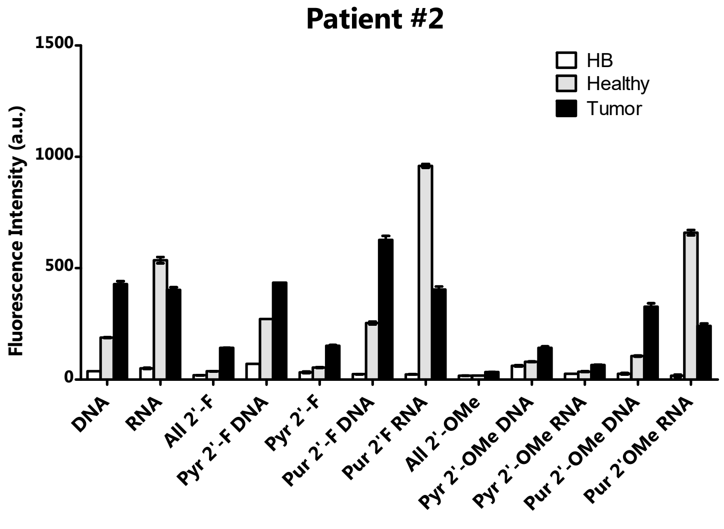

Figure S1. Representative raw data for the screening of nuclease activity in breast tissue biopsies. Increase in fluorescence intensity is correlated with nuclease degradation activity of paired samples: Healthy (**grey bars**) and tumor (**black bars**) tissue samples from two different patients. HB (homogenization buffer) was used as control, to correct for the probe background signal. Bars represent the average ± s.d. of triplicate measurements.

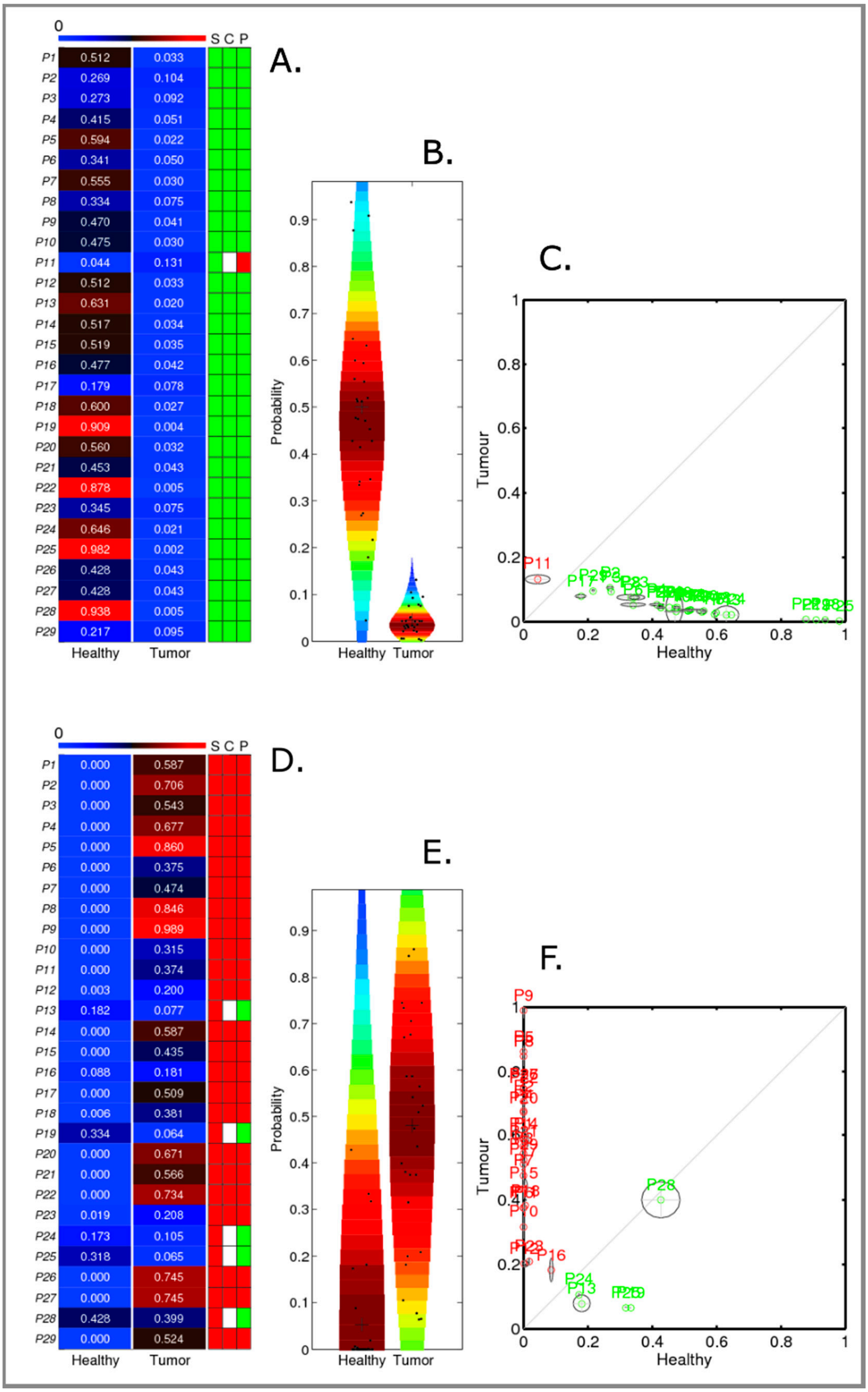

Figure S2. Predictions with the combination of probes {p01, p04, p06} of the healthy and tumor samples from the retrospective screening #1. Prediction of healthy samples is visualized in **A**, **B** and **C**. Prediction of tumor samples is visualized in D, E and F. (**A**,**D**) Heatmaps of the probabilities of each patient sample to be healthy or tumor. The table to the right marks: S, real status of the sample,

P, prediction of the status, and C the comparison between them. Healthy in green, tumor in red, and mismatch between real status and prediction in white. (**B**,**E**) Violin plots of the distribution of the estimated probability for each group (healthy or tumor). The red squares represent the position of the means. The blue points represent the spread of the probability of the probe intensities used to build the distributions. (**C**,**F**) Scatter plots of the predicted probabilities of belonging to the tumor group versus the healthy group. Healthy in green, tumor in red. The axes of the ellipses are proportional to the standard deviation of the probability prediction of the probes. Pi, patient i.

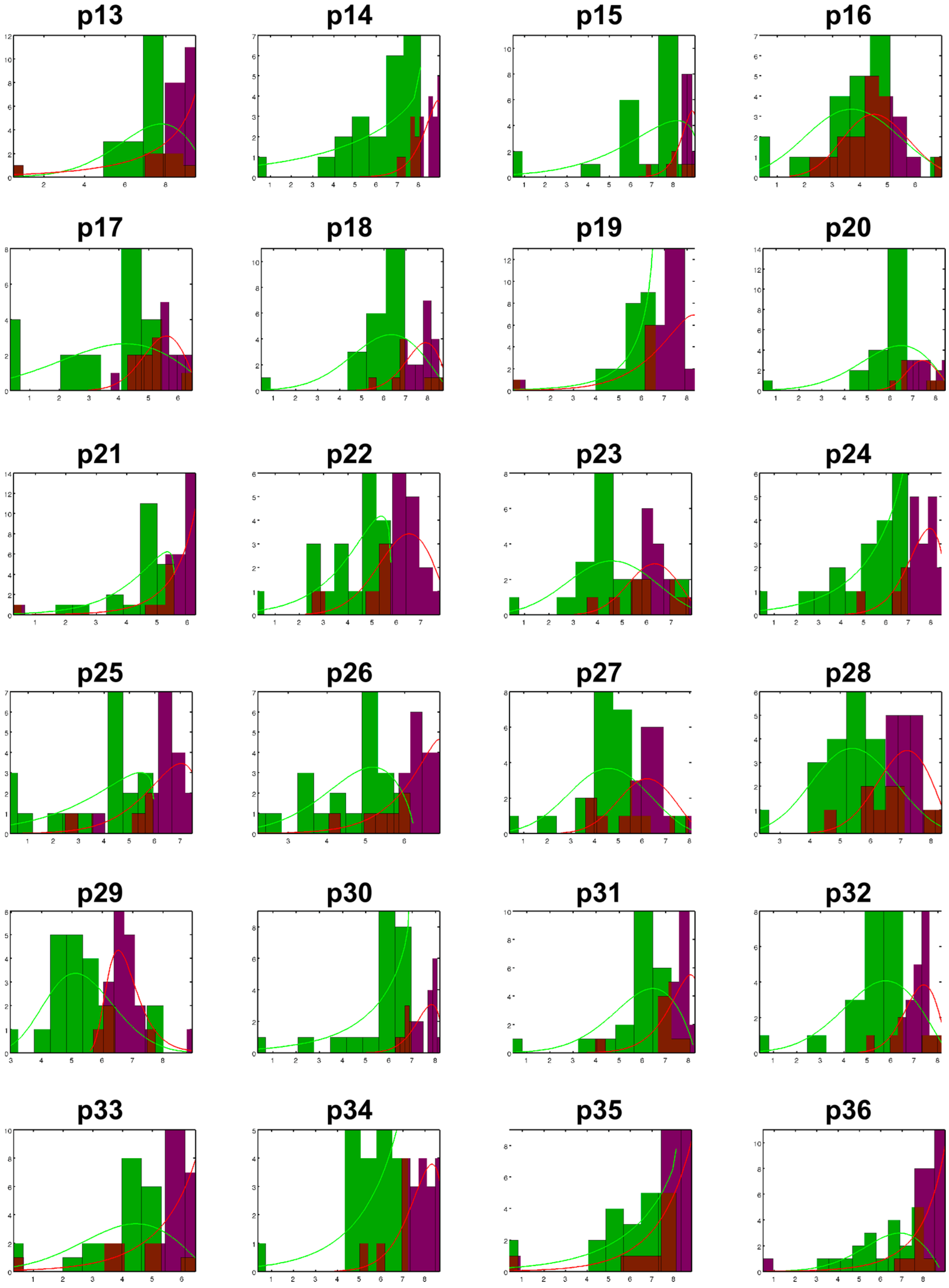

Figure S3. Empirical distribution functions of 24 additional probes on the retrospective screening #2. Histograms and empirical distribution functions of the probes in healthy (**green**) and tumor (**red**) samples for the identification of best performing probes. The empirical distribution functions are

represented by the continuous lines in green and red for healthy and tumor samples, respectively. Less overlap between distributions corresponds to better discrimination between healthy and tumor samples.

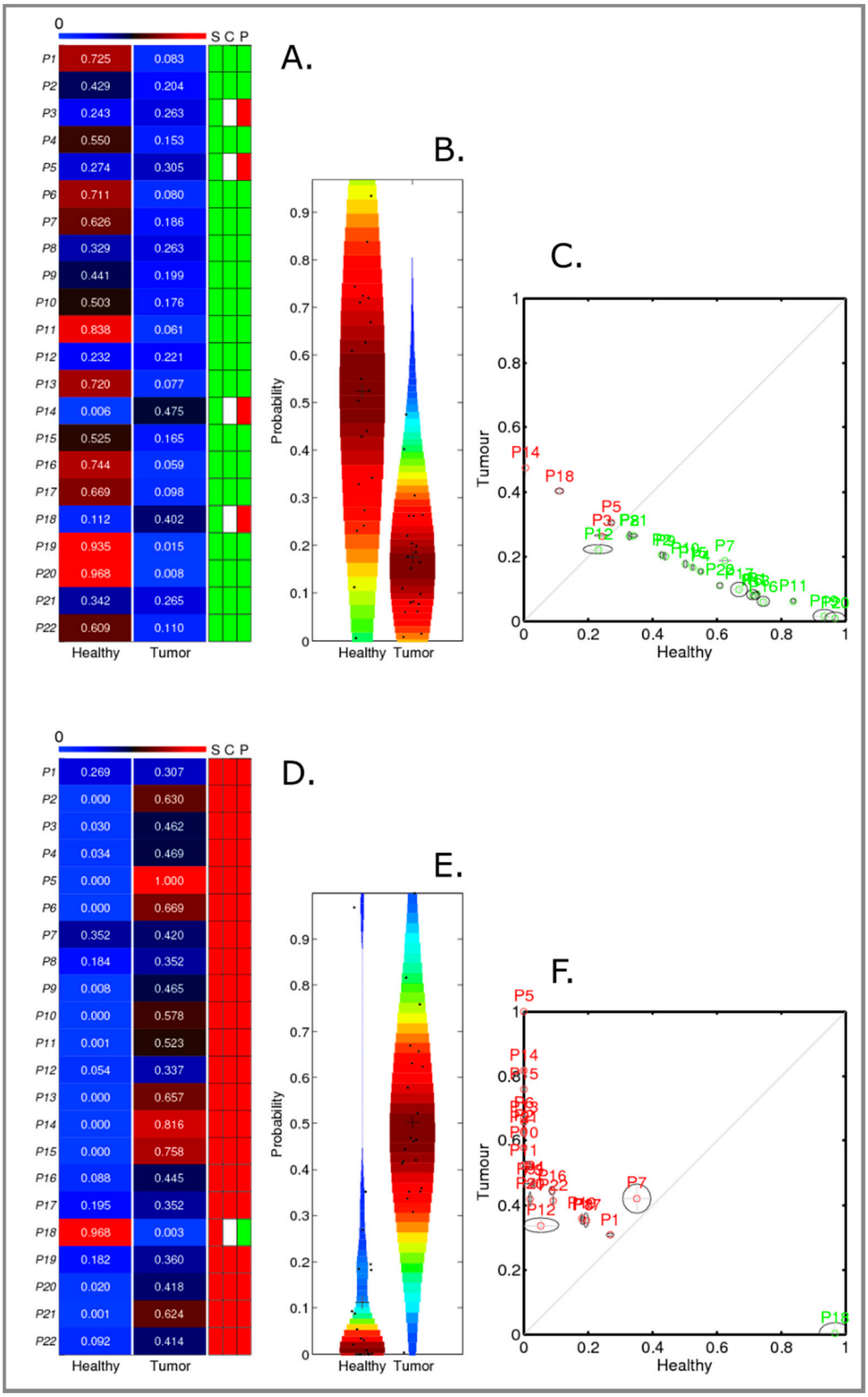

Figure S4. Predictions with the combination of probes {p13, p35, p36} of the healthy and tumor samples from the retrospective screening #2. Prediction of healthy samples is visualized in **A**, **B** and **C**. Prediction of tumor samples is visualized in D, E and F. (**A**,**D**) Heatmaps of the probability of each sample to be healthy or tumor. The table to the right marks: S real status of the patient sample, P

prediction of the status, and C, the comparison between them. Healthy in green, tumor in red, and mismatch between real status and prediction in white. (**B**,**E**) Violin plots of the distribution of the estimated probability for each group (healthy or tumor). The red squares represent the position of the means. The blue points represent the spread of the probability of the probe intensities used to build the distributions. (**C**,**F**) Scatter plots of the predicted probabilities of belonging to the tumor group versus the healthy group. Healthy in green, tumor in red. The axes of the ellipses are proportional to the standard deviation of the probability prediction of the probes. Pi denotes patient i.

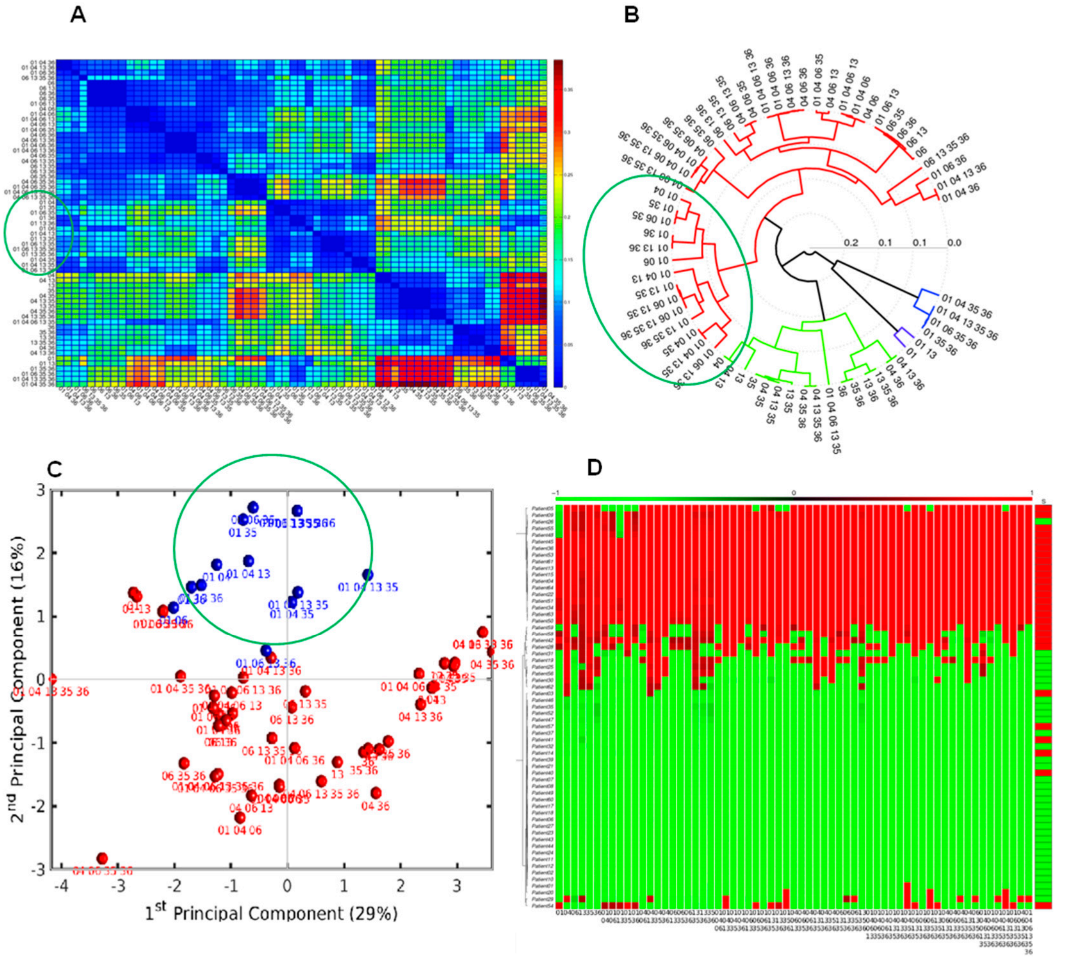

Figure S5. Search of the optimal combination of the six {p01, p04, p06, p13, p35, p36} predictor variables for the discrimination analysis between healthy and tumor on the prospective dataset. (**A**) Map of the distances of probability of predictions achieved between combinations of predictors. The color bar gives a color codification of the distances. The closer the predictions between two combinations of predictors are, the bluer the color and, conversely, the further they are the redder the color. (**B**) Circular hierarchical clustering of combination of predictors performed using the correlation metric and the average linkage method. (**C**) Principal Component Analysis (PCA) of all possible combinations of predictors based on their prediction probabilities. The green ellipse encloses a group of combinations of probes (in blue color) that have close good prediction features to all other combinations of probes. (**D**) Heatmap of the prediction of all the combinations of predictor variables for the discriminant analysis between healthy and tumor. The prediction of healthy and tumor states is depicted in negative probabilities (**in green**) and positive probabilities (**in red**). The table to the right marks real state, S, of the sample (healthy in green, tumor in red).

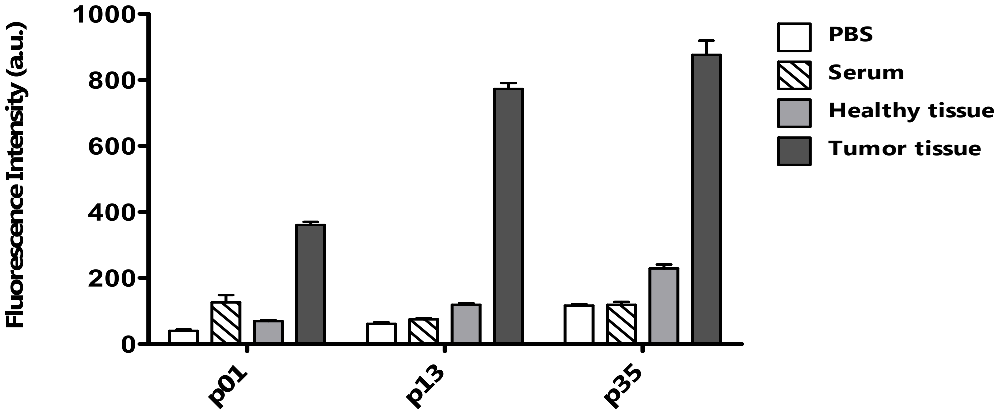

Figure S6. Serum stability of the three "cancer probes". The probes p01, p13 and p35 were tested for nuclease activity in human serum (**hashed bars**), healthy tissue homogenate (**light grey**) and tumor tissue homogenate (**dark grey**). PBS (**white bars**) was used as control in these experiments. The bars represent average fluorescent intensity measurements of three individual experiments. Error bars represent the standard deviation from the mean.

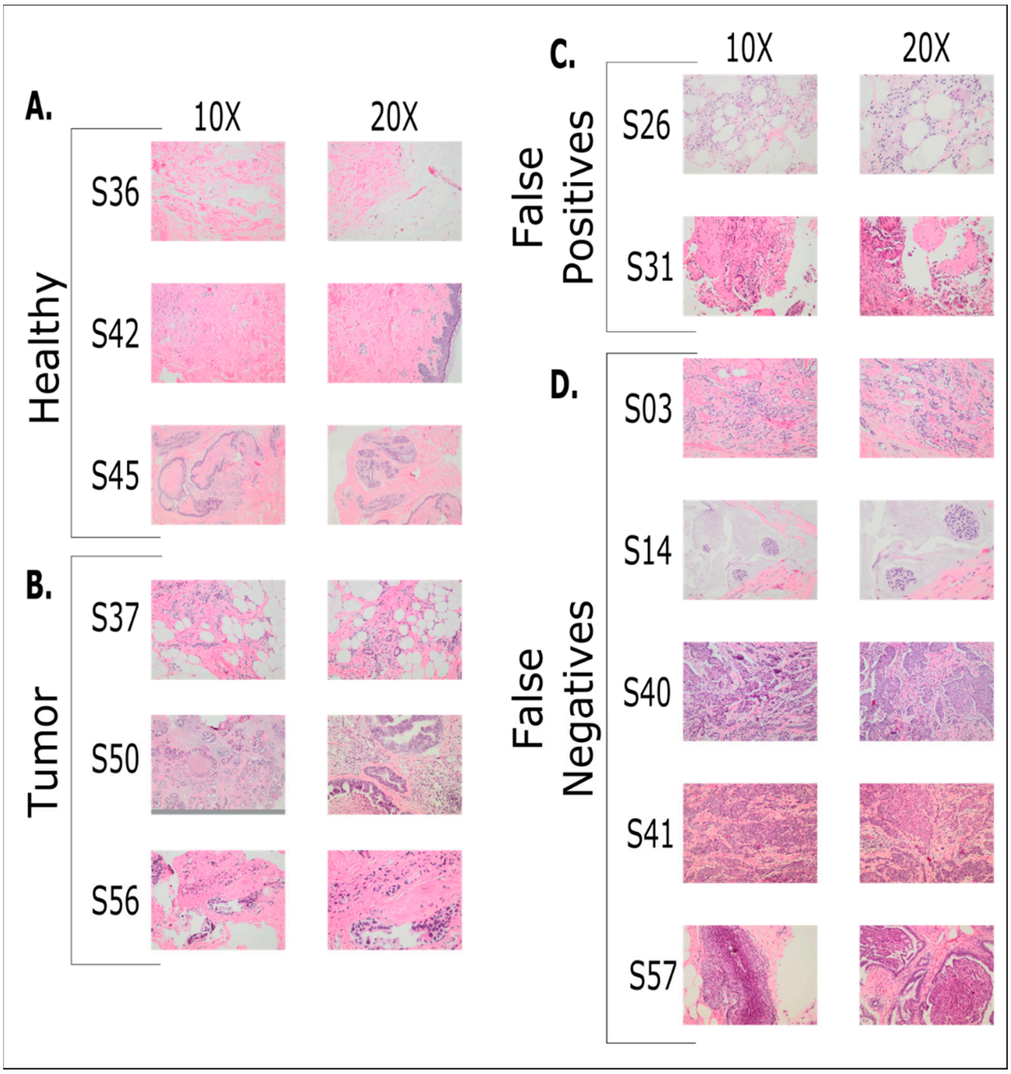

Figure S7. Hematoxylin and Eosin (H&E) staining of representative samples of breast tissues. (**A**) Healthy breast tissue: with the presence of connective and adipose tissue in P36, post-mastectomy

scar skin with epidermis and dermis next to the lesion in P42, and fibroepithelial lesion with obliteration of glandular structures and focal adenosis in P45. (**B**) Tumor tissue: invasive neoplasia with glandular forms and carcinoma in situ with presence of central necrosis in P32, invasive tumor cells between adipocytes and the surroundings in P37, tumor cells showing pleomorphism with invasive and in situ component. (**C**) False positives: inflammatory granulomatous lesion in response to foreign body, macrophages and multinucleated giant cells are observed in both P26 and P31. (**D**) False negatives: tumor tissue with predominance of glandular invasive component in P03, mucinous component in P14, tumoral solid pattern in P40 and P41, and benign papillary lesion with *in situ* neoplasia in P57. All samples are 4 µm sections. All images were acquired using 10× and 20× objectives, as indicated in the pictures.

Table S1. List of oligonucleotide probes used in this study. Nucleic acid probes containing natural and chemically modified nucleotides were designed and used as substrates in assaying nuclease activity associated with healthy and tumor samples.

Sequence Name	Sequence
DNA (p01**)**	FAM//TCTCGTACGTTC//TQ2
RNA (p02)	FAM//ucucguacguuc//TQ2
All 2′-F (p03)	FAM//fUfCfUfCfGfUfAfCfGfUfUfC//TQ2
Pyr 2′-F DNA (p04)	FAM//fUfCfUfCGfUAfCGfUfUfC//TQ2
Pyr 2′-F RNA (p05)	FAM//fUfCfUfCgfUafCgfUfUfC//TQ2
Pur 2′-F DNA (p06)	FAM//TfAfACfGTfACfGfGTC//TQ2
Pur 2′-F RNA (p07)	FAM//ufCufCfGuafCfGuufC//TQ2
ZAll 2′-OMe (p08)	FAM//mUmCmUmCmGmUmAmCmGmUmUmC//TQ2
Pyr 2'-OMe DNA (p09)	FAM//mTmCmTmCGmTAmCGmTmTmC//TQ2
Pyr 2'-OMe RNA (p10)	FAM//mUmCmUmCgmUamCgmUmUmC//TQ2
Pur 2′-OMe DNA (p11)	FAM//TmAmACmGTmACmGmGTC//TQ2
Pur 2′-OMe RNA (p12)	FAM//umAmAcmGumAcmGmGuc//TQ2
Poly A 2′-F DNA (p13)	FAM//fAfAfAfAfAfAfAfAfAfAfAfA//TQ2
Poly C 2′-F DNA (p14)	FAM//fCfCfCfCfCfCfCfCfCfCfCfC//TQ2
Poly U 2′-F DNA (p15)	FAM//fUfUfUfUfUfUfUfUfUfUfUfU//TQ2
A chi DNA (p16)	FAM//mUmUmCmUmCmCmUfAmUmCmCmUmCmUmU//TQ2
AA chi DNA (p17)	FAM//mUmUmCmUmCmCmUfAfAmUmCmCmUmCmU//TQ2
AAA chi DNA (p18)	FAM//mUmCmUmCmCmUfAfAfAmUmCmCmUmCmU//TQ2
CCC chi DNA (p19)	FAM//mUmCmUmCmCmUfCfCfCmUmCmCmUmCmU//TQ2
UUU chi DNA (p20)	FAM//mUmCmUmCmCmUfUfUfUmUmCmCmUmCmU//TQ2
GGG chi DNA (p21)	FAM//mUmCmUmCmCmUfGfGfGmUmCmCmUmCmU//TQ2
AAC chi DNA (p22)	FAM//mUmCmUmCmCmUfAfAfCmUmCmCmUmCmU//TQ2
ACA chi DNA (p23)	FAM//mUmCmUmCmCmUfAfCfAmUmCmCmUmCmU//TQ2
CCA chi DNA (p24)	FAM//mUmCmUmCmCmUfCfCfAmUmCmCmUmCmU//TQ2
CAC chi DNA (p25)	FAM//mUmCmUmCmCmUfCfAfCmUmCmCmUmCmU//TQ2
AAU chi DNA (p26)	FAM//mUmCmUmCmCmUfAfAfUmUmCmCmUmCmU//TQ2
AUA chi DNA (p27)	FAM//mUmCmUmCmCmUfAfUfAmUmCmCmUmCmU//TQ2
UUA chi DNA (p28)	FAM//mUmCmUmCmCmUfUfUfAmUmCmCmUmCmU//TQ2
UAU chi DNA (p29)	FAM//mUmCmUmCmCmUfAfAfUmUmCmCmUmCmU//TQ2
CUC chi DNA (p30)	FAM//mUmCmUmCmCmUfCfUfCmUmCmCmUmCmU//TQ2
UCU chi DNA (p31)	FAM//mUmCmUmCmCmUfUfCfUmUmCmCmUmCmU//TQ2
AGA chi DNA (p32)	FAM//mUmCmUmCmCmUfAfGfAmUmCmCmUmCmU//TQ2
GAG chi DNA (p33)	FAM//mUmCmUmCmCmUfGfAfGmUmCmCmUmCmU//TQ2
AAA AAA chi DNA (p34)	FAM//mUmCmUfAfAfAmCmUmCfAfAfAmUmCmU//TQ2
AAA CCC chi DNA (p35)	FAM//mUmCmUfAfAfAmCmUmCfCfCfCmUmCmU//TQ2
AAA UUU chi DNA (p36)	FAM//mUmCmUfAfAfAmCmUmCfUfUfUmUmCmU//TQ2

Uppercase TACG = DNA, Lowercase uacg = RNA, m = 2′-O-Methyl modification, f = 2′-Fluoro modification.

Table S2. Patient histopathological diagnosis (prospective study).

Patient ID #	Histopathological Diagnostic
Patient 01	Normal benign
Patient 02	Normal benign
Patient 03	Malignant (IDC)
Patient 04	Malignant (IDC + DCIS)
Patient 05	Malignant (IDC)
Patient 06	Benign (radial scar and hyperplasia)
Patient 07	Normal benign
Patient 08	Benign, inflammatory
Patient 09	Malignant (IDC + DCIS)
Patient 10	Normal benign
Patient 11	Normal benign
Patient 12	Normal benign
Patient 13	Malignant (Papillar carcinoma)
Patient 14	Malignant (Mucinous carcinoma)
Patient 15	Malignant (IDC)
Patient 16	Eliminated from the study
Patient 17	Normal benign
Patient 18	Normal benign (inflammation)
Patient 19	Normal benign (inflammation)
Patient 20	Normal benign
Patient 21	Normal benign
Patient 22	Malignant (Adenoid cystic carcinoma)
Patient 23	Benign, fibrosis
Patient 24	Normal benign
Patient 25	Normal benign
Patient 26	Benign, granulomatous reaction to silicon
Patient 27	Normal benign
Patient 28	Malignant (IDC)
Patient 29	Benign
Patient 30	Benign (tissue adjacent to silicon implant, silicon granulomatosis)
Patient 31	Post-surgical granulomatous reaction
Patient 32	Benign (no tumor, columnar metaplasia)
Patient 33	Eliminated from the study
Patient 34	Malignant (Invasive ductal carcinoma (IDC), G2)
Patient 35	Normal (adipose fibrous tissue)
Patient 36	Malignant (IDC, G2)
Patient 37	Normal benign
Patient 38	Eliminated from the study
Patient 39	Normal benign
Patient 40	Malignant (ductal carcinoma in situ DCIS, only one tumoral duct)
Patient 41	Malignant (IDC, G2)
Patient 42	Malignant (IDC, G2)
Patient 43	Normal (skin with mild inflammation)
Patient 44	Normal
Patient 45	Malignant (IDC, G2)
Patient 46	Benign (Fibroadenoma and adenosis)
Patient 47	Benign
Patient 48	Malignant (IDC, G2 with lesion from previous biopsy)
Patient 49	Normal (adipose tissue)
Patient 50	Matastatic ganglion and breast IDC

Patient 51	Malignant (IDC, G1 + IDC)
Patient 52	Normal and fibroadenoma
Patient 53	Malignant (IDC, G2)
Patient 54	Malignant
Patient 55	Malignant (IDC, G2)
Patient 56	Benign (inflammation and hyperplasia)
Patient 57	Malignant (Papilloma with adjacent DCIS, poorly represented)
Patient 58	Malignant
Patient 59	Malignant
Patient 60	Benign
Patient 61	Malignant (IDC, G3)
Patient 62	Benign
Patient 63	Malignant (IDC, G2)
Patient 64	Malignant (IDC with DCIS)

Table 3. Contingency table of the discriminant analysis using the best combination of probes {p01, p13, p35}. Sensitivity, specificity, positive predictive value and negative predictive value for the combination of the cancer probes are included in the table and calculated using the equations below.

	**Tumor**	**Healthy**	**Predicted Condition**
Positive (number)	True Positives (A) 22	False Positives (B) 2	Predicted Positive 24
Negative (number)	False Negatives (C) 5	True Negatives(D) 32	Predicted Negative 37
Total (number)	Total Tumor 27	Total Healthy 34	Total 61

Sensitivity: A/(A + C) × 100 = 22/ (22 + 5) × 100 = 82%, Specificity: D/(D + B) × 100 = 32/ (32 + 2) × 100 = 94%, Positive Predictive Value: A/(A + B) × 100 = 22/ (22 + 2) × 100 = 92%, Negative Predictive Value: D/(D + C) × 100 = 32/ (32 + 5) × 100 = 87%.

© 2021 by the authors. Licensee MDPI, Basel, Switzerland. This article is an open access article distributed under the terms and conditions of the Creative Commons Attribution (CC BY) license (http://creativecommons.org/licenses/by/4.0/).
